# Changes in Recognition Memory over Time: An ERP Investigation into Vocabulary Learning

**DOI:** 10.1371/journal.pone.0072870

**Published:** 2013-09-05

**Authors:** Shekeila D. Palmer, Jelena Havelka, Johanna C. van Hooff

**Affiliations:** 1 University of York, Department of Psychology, York, North Yorkshire, United Kingdom; 2 University of Leeds, Department of Psychology, Leeds, West Yorkshire, United Kingdom; 3 VU University Amsterdam, Department of Psychology, Amsterdam, Netherlands; University of Goettingen, Germany

## Abstract

Although it seems intuitive to assume that recognition memory fades over time when information is not reinforced, some aspects of word learning may benefit from a period of consolidation. In the present study, event-related potentials (ERP) were used to examine changes in recognition memory responses to familiar and newly learned (novel) words over time. Native English speakers were taught novel words associated with English translations, and subsequently performed a Recognition Memory task in which they made old/new decisions in response to both words (trained word vs. untrained word), and novel words (trained novel word vs. untrained novel word). The Recognition task was performed 45 minutes after training (Day 1) and then repeated the following day (Day 2) with no additional training session in between. For familiar words, the late parietal old/new effect distinguished old from new items on both Day 1 and Day 2, although response to trained items was significantly weaker on Day 2. For novel words, the LPC again distinguished old from new items on both days, but the effect became significantly larger on Day 2. These data suggest that while recognition memory for familiar items may fade over time, recognition of novel items, conscious recollection in particular may benefit from a period of consolidation.

## Introduction

Following a single learning episode the quantity of information that can be recalled typically decreases over time. In fact, the rate of forgetting in empirical investigations is remarkably predictable, occurring rapidly over the first few hours, before slowly levelling off [1]. Although forgetting may be perceived as a limitation on cognitive performance, it is likely that it provides some adaptive function. As new input is continually acquired, memory must be constantly updated and modified. If old information did not decay then it may compete or interfere with the processing of new information [2]. In order to retain relevant long-term knowledge however, the brain must be able to counteract decay. While it is well known that certain strategies such as intentional rehearsal can help maintain memories, it is also thought that memory consolidation can occur automatically, without conscious effort or intent [3].

In recent years memory retention over time has been investigated primarily in the context of sleep studies. This work has been motivated by the widely held belief that sleep provides optimal conditions for offline memory consolidation [4]. Indeed many studies have demonstrated that participants display superior recall performance when the retention interval between learning and test is filled with sleep rather than wakefulness [e.g. 5–7]. Findings relating to recognition memory have been less consistent with studies reporting only modest effects, or even no effects of sleep at all on overall recognition performance [4].

Some of this inconsistency may stem from the fact that recognition memory is not a unitary process, and that different aspects of recognition memory may be differentially affected by consolidation processes. According to dual process accounts, recognition is supported by two distinct mechanisms known as familiarity and recollection (see Yonelinas [8] for a review). Familiarity is described as a relatively fast and automatic ‘sense of knowing’ which occurs without recall of any qualitative contextual information about the study episode. Recollection on the other hand is a slower and more effortful process in which explicit contextual information associated with prior exposure to the stimulus becomes available [9]. In the context of sleep research, studies by Daurat et al. [10], using the Remember/Know paradigm, and Drosopoulos et al. [11] using a process dissociation procedure, have both reported that sleep benefits recollection, but has no effect on familiarity judgements. This indicates that the way in which consolidation processes operate on recognition memory may be more qualitative than quantitative in nature.

While these studies demonstrate that different activities performed during the retention interval can have differential effects on memory, one issue that is seldom addressed is how memories have *changed* over time. More specifically, due to the lack of an initial recall or recognition test on the same day as the leaning episode, there is little data on how older memories compare to their initial state after learning. Studies which have included such baseline test have observed an overall decline in performance over time, but note that the decline is less severe in the sleep condition [e.g. 10]. This research suggests therefore that sleep has a preservative function, in that it is associated with a reduction in the proportion of loss over time compared to equivalent periods of wakefulness.

The present investigation was primarily concerned not with the quantity of information recalled, but with the qualitative changes associated with items that are recalled/recognised following a period of consolidation. Event-related potentials (ERPs) provide one means of examining the amount of memory activation elicited by a given stimulus, and the relative contributions of the different processes underlying recognition memory judgements. ERPs are time locked voltage changes which occur in the brain in response to a specific stimulus. A large body of research has reliably demonstrated that ERPs elicited by items correctly recognised as ‘old’ (i.e. previously appearing in a study phase) are more positive than those elicited by correctly rejected ‘new’ (not studied) items around 300–800 ms following target onset. Within this timeframe two separate old/new effects have been identified which differ in latency and scalp distribution. The most apparent of these effects occurs around 400–800 ms and is maximal at left parietal electrode positions. This posterior old/new effect is likely related to the late positive component (LPC) and is generally believed to reflect conscious recollection [9], [12]–[15]. It is preceded and slightly overlaps with a less pronounced old/new effect which is typically more prominent over frontal areas. This early old/new effect, occurring around 300–500 ms, is believed to be associated with the N400 and is presumed to reflect familiarity [9], [12]–[15], though this is somewhat debated [16].

To date, very few ERP studies have examined how the neural correlates of recognition memory change over time. In two recent reports [17], [18] participants studied a large number of faces before undergoing a retention interval filled with either sleep or their normal waking activities. ERPs were then recorded while participants performed an old/new recognition test. Consistent with the view that sleep has a preservative effect on memory, the amplitude difference between old and new items was larger when the retention interval was filled with sleep rather than wakefulness. Importantly, in support of results from behavioural studies [10], [11], this effect was only evident in the LPC associated with conscious recollection. However, as explained before, given that neither study included an initial baseline test immediately after learning, it is still not known how recognition memory changed over the retention interval (i.e. whether LPC effects became smaller in the wake condition or larger in the sleep condition).

One ERP study which has addressed this issue compared recognition memory correlates at different retention intervals [19]. Participants studied 192 faces before being tested for recognition at four different delay intervals (after half an hour, one hour, one day and one week). An interaction was observed between stimulus status and delay during the LPC time window indicating that the old-new difference was significant only at the one day and one week delay intervals. No such interaction was reported in the earlier time windows. The authors suggested that this interaction may be linked to processes of memory consolidation as the newly acquired information requires time to be firmly encoded into long term memory. However it is unclear why no significant old/new effects at all were observed at the shorter retention intervals, since this seems to go against results of the majority of other studies in which significant old/new effects have been observed immediately after the study phase [e.g. 12, 15, 20]. A possible reason for this discrepancy is that participants in Joyce and Kutas' [19] study were presented with a very large number of stimuli during the learning phase, and some of these were presented for a very brief duration (300–1000 ms). It is possible therefore that the memory traces for individual items were initially very weak, and became stronger over time as a result of consolidation.

The idea that recognition memory actually increases in strength over time seems somewhat at odds with behavioural data from sleep studies which indicate a pattern of overall loss over time. However, it has been argued that effects of consolidation are stronger when the memory trace is initially weak [4], which was clearly the case in Joyce and Kutas' [19] study where memory traces were initially so weak that they failed to produce any significant old/new ERP effects on the same day as learning. An alternative possibility is that recognition memory for familiar and novel information may be differentially affected by length of time between study and test. Unlike the behavioural studies by Daurat et al. [10] and Drosopoulos et al. [11] which used word stimuli that were already familiar to participants prior to the learning phase, the ERP studies mentioned above all used face stimuli that were entirely novel to participants prior to learning. Indeed it is likely that the consolidation process associated with memory for known and novel items is quite different.

According to the complimentary learning systems account of consolidation [21], new declarative memories are encoded by both the hippocampal and the neocortical system. The role of the hippocampal formation is to bind together relevant areas of the neocortex via a process of repeated co-activation, which results in long-lasting modification of the connections between the cortical areas. McClelland et al. [21] argue that reactivation can occur either in task relevant situations in which the memory trace is required for task performance or in off-line situations such as during sleep. The hippocampal system therefore acts as a temporary store for new memories which allows them to be integrated into the neocortex in a gradual way. Since novel items have no existing long-term representation, it is likely that they will initially rely more heavily on hippocampal storage and consolidation processes in order to create a new representation. In other words, new memory traces for novel items are likely to require more extensive system-level reorganisation. In contrast, items which are already familiar to the participant prior to learning will already have existing long-term representations in the neocortex. It is therefore episodic information associated with a particular encounter which is important for recall/recognition. However, according to McClelland et al. [21], when an event that is reinstated repeatedly in different contexts, the accumulated changes to neocortical connections are most likely to preserve common aspects of the reinstated event, and specific contextual information that is not repeated may not be well maintained. We hypothesise therefore that any evidence of consolidation will be more prominent for novel information, relative to that what is already known.

The present investigation sought to examine changes in recognition memory for novel and familiar words in a typical learning scenario which was designed to resemble second language vocabulary acquisition. Participants were trained on 28 translation pairs consisting of a novel word form paired with an English translation. Since our primary interest was in qualitative changes associated with items that are correctly remembered, participants underwent an extensive training phase in which they were presented with corrective feedback cycles until they were able to attain 100% accuracy on recognition and production measures for all items. Following training, ERPs were recorded while participants performed an old/new recognition task on both novel word forms and the familiar English words (Day1). Given the evidence that sleep is necessary for optimal consolidation, participants came back to the lab and repeated the old/new recognition task the following day with no additional training (Day 2). Episodic memory responses to novel and familiar word forms were compared across days to examine how memory changed over time. Given that previous behavioural studies have found that sleep benefits recollection but not familiarity, it is hypothesised that any evidence of memory consolidation would be manifested primarily in the late, parietal old/new effect.

## Method

### Participants

Twenty five undergraduate students (22 female, 3 male) from the University of Kent participated in this experiment in return for course credit. All were native English speakers with normal or corrected vision. Participant ages ranged from 18 to 22 (mean age 19.36 years) and all were right handed. Since modern languages are taught in most schools throughout England, most participants had some previous experience in foreign language learning. However, none reported that they were able to speak a second language fluently. Ethical approval was obtained from the departmental ethics committee at the University of Kent. Participants were informed on all study procedures and gave written consent before taking part in this investigation.

### Stimuli and Design

The experiment consisted of three parts; 1) a training phase during which participants were required to learn 28 novel words along with their corresponding English translations, 2) an Episodic Memory task performed approximately 45 minutes after the training phase (Day1), 3) a repeat of the Episodic Memory task performed the following day (Day 2). The Episodic Memory task was of a 2×2×2 within participants design with day (Day 1 vs. Day 2), training (trained item vs. untrained item), and word type (English word vs. novel word) as the independent variables.

Fifty six English words were selected for the experiment, 14 from each of four semantic categories; 1) clothing, 2) animals, 3) body parts and 4) vehicles. These 56 words were split into two sets of 28 words (subsequently referred to as ‘set A’ and ‘set B’), with each set including seven words from each of the four categories. Within each semantic category, words allocated to set A and set B were matched for frequency (clothing, *t*(12) = 0.01, *p* = 1.00; animals, *t*(12) = −18, *p* = 86; body parts, *t*(12) = −13, *p* = 90; transport, *t*(12) = 20, *p* = 84) and length (clothing, *t*(12) = 0.0, *p* = 1.00; animals, *t*(12) = −1.102, *p* = 29; body parts, *t*(12) = −229, *p* = 82; transport, *t*(12) = 0.00, *p* = 1.00). Word length in each set ranged from 3–9 letters (set A mean = 4.96; set B mean = 4.93). The mean word frequency was 1043.21 (SD  = 1608.71) in set A and 1064.64 (SD  = 1599.60) in set B (frequency ratings taken from Celex Lexical Database, [22]. Each of the 56 English words was paired with a ‘novel’ word. The novel words were all nonwords which conformed to the phonotactic constraints of English and they matched the English words in length (for examples, see Appendix S1). The 56 novel words and their corresponding English translations were termed ‘translation pairs’ for the purpose of this experiment.

During the Episodic Memory task trained items consisted of 28 novel words and the corresponding English translations that the participant had learned during the training phase (either set A or set B, counterbalanced between participants). The untrained items consisted of the 28 Novel words and the corresponding English translations from the set that the participants had not been exposed to during the training phase. All participants therefore experienced four trial types during the Episodic Memory Task; 1) 28 trained English words, 2) 28 trained novel words, 3) 28 untrained English words, 4) 28 untrained novel words.

### Procedure

#### The training phase

The main goal of the training phase was to ensure that all participants were trained on items to a similar standard. A programme was designed which tested participants on items in a number of stages, and participants were required to attain 100% accuracy on each stage before moving onto the next. The training programme was constructed using E-Prime software and all participants were trained individually in laboratory conditions. Participants were required to complete the programme on Day 1 before performing the Episodic Memory task.

During training, half of the participants learnt the word pairs in set A and half learnt set B. Participants learnt the 28 word pairs in four blocks of seven. The four blocks corresponded to the four semantic categories (clothing, animals, body parts and vehicles). The order in which these blocks were learnt was counterbalanced between participants. In order to learn the words in each block participants had to complete four stages as follows:

*Stage 1:* Participants were simply required to view the seven novel words with their English translations and try to memorise each word pair as it appeared. Each word pair was displayed on a computer screen for 4000 ms preceded by a fixation cross lasting 500 ms. All words were presented in lowercase letters and each pair was separated by a hyphen in the centre of the screen. The English word would appear on the left hand side of the hyphen and the novel word would appear on the right (e.g. hat – bem). A blank screen lasting for 1000 ms followed the presentation of each word pair. After the participants had viewed all seven word pairs once, the programme moved on to stage 2.

*Stage 2:* During stage 2 the seven word pairs were presented again, but this time participants were required to type each word pair immediately after it had been presented. Each word pair was presented for 3000 ms preceded by a fixation cross lasting 500 ms. This time, instead of a blank screen, a text entry screen followed each word pair. Participants would see only a hyphen in the centre of the screen and were required to type in the word pair that they had just seen, followed by a press on the return key. If the participant had typed the words correctly, then the programme would move on to the next word pair. However, if the participant made a mistake in the text entry, then the same word pair was presented again for 3000 ms followed by the text entry screen. This sequence would repeat until the participant had typed both words in the pair correctly. Once the participant had typed all seven word pairs correctly the programme would proceed to stage 3.

*Stage 3:* During stage 3 participants would view the seven novel words and were required to type the English translations from memory. During each trial a fixation cross appeared for 500 ms, followed by a hyphen appearing in the centre of the screen with one of the novel words presented on the right. Participants were required to type the corresponding English translation into the blank space on the left and then press return key. If the participant typed the correct translation then the programme would move on to the next trial. If the participant typed the translation incorrectly, then the correct word pair would appear on the screen for 3000 ms before the programme moved on to the next trial. Once the participant had completed all seven trials, stage 3 would be repeated (with a different word order), unless the participant had achieved 100% accuracy.

*Stage 4:* Stage four was almost identical to stage 3 except that this time participants would view the seven English words and were required to type the associated novel word from memory. During each trial a fixation cross appeared for 500 ms, followed by a hyphen appearing in the centre of the screen with one of the English words presented on the left. Participants were required to type the corresponding novel word translation into the blank space on the right and then press return key. As in stage 3, the participant received corrective feedback and repeated the cycle until they had typed all seven translations correctly in a single cycle.

Once stage 4 had been completed participants were asked to take a short break and press the space bar when they were ready to continue. Once the participant indicated that they were ready, stages 1 to 4 would be repeated for the next block of seven word pairs. After all four blocks of seven words had been learned participants completed two final stages which required them to recall all 28 word pairs that they had learned.

*Stage 5:* The first of these two stages was almost identical to stage 3 except that participants were required to type the English translations for all 28 new words, rather than just seven. The 28 words were presented in a random order and if the participant entered any translations incorrectly, then they would receive corrective feedback, and at the end of the cycle, be required to type all 28 words again. Each time the cycle repeated E-Prime would scramble the presentation order of the 28 words. However, in order to support the learning of any particular items that the participant was struggling to remember, before the cycle repeated the participant would be re-tested on the words that they had typed incorrectly during the previous cycle. The cycle would continue to repeat until participants typed all 28 English translations correctly. At the end of stage 5 participants were asked to take a short break before moving on to stage 6.

*Stage 6:* The final stage was identical to the previous one, except that participants were presented with the English words and were required to produce the 28 corresponding nonwords. As in stage 4, the English words would appear on the left hand side of the hyphen and participants were required to type the new translation into the blank space on the right. The training session ended once the participant had typed all 28 translations correctly in a single cycle.

Throughout the training session the E-Prime software recorded data on the number of cycles that it took for each participant to type all words correctly during stages 3–6, and on the number of errors made during each cycle. The length of time taken to complete the training phase varied between participants but the majority managed to complete it within 45 minutes.

#### Episodic Memory Task – Day 1

Once participants had completed the training phase electrodes were applied before the participants performed the Episodic Memory task for the first time. The electrode application took approximately 45 minutes and during this time the participant was encouraged to relax. Each trial consisted of a fixation cross (500 ms) followed by one of the stimuli appearing in black lowercase lettering in the centre of a white screen. Participants were instructed to indicate whether or not they had seen the item during the training phase of the experiment by pressing Z or M (for yes or no, counterbalanced between participants) on the computer keyboard. The stimulus remained on the screen until the participant responded and a blank screen lasting 1000 ms separated each trial. Stimuli were presented in a pseudorandom order, the randomisation constraint being that translation equivalents could not follow one another. In addition to the electrophysiological recording, response times and accuracy were recorded by the E-Prime software. In total the task lasted approximately 10 minutes.

#### Episodic Memory Task – Day 2

On Day 2 participants returned to the laboratory, electrodes were reapplied and the participant repeated the Episodic Memory task with no additional training session.

### Electrophysiological measures

During the Episodic Memory tasks the EEG was recorded from 19 Ag/AgCl electrodes (average reference). Electrode locations were based on the standard international 10–20 system including seven frontal (Fz Fp1, Fp2, F2, F3, F7, F8), three central (Cz, C3, C4), two temporal (T7, T8), five parietal (Pz, P3, P4, P7, P8) and two occipital (O1, O2) electrodes embedded in a nylon EEG cap. The ground electrode was Fpz and clip-on electrodes recorded activity from the earlobes (A1 and A2). Vertical electro-oculogram (VEOG) activity was recorded from electrodes placed above and below the left eye. EEG signals were amplified using a band pass filter of 0.01–25 Hz and digitised online at sampling frequency of 250 Hz using a 16 bit A/D converter. Brain Vision recording software (version 1.02) was used with a Quickamp 72 amplifier. Electrode impedances were kept below 5 kΩ.

The EEG data was corrected for eye movements off-line using the Gratton and Coles [23] method, as implemented in the Brain Vision analysis software. The data was also screened for recording artefacts. Artefact rejection was based on the following criteria: a) maximum allowed voltage step of 50 µV between two sample points, b) maximum allowed absolute difference of 80 µV over a 200 ms interval, and c) lowest allowed activity of 0.5 µV over a 100 ms interval. The recordings were re-referenced to the earlobes (A1 and A2) and trials were segmented from 100 ms prior to word onset to 1400 ms after word onset. The baseline interval was defined as –100 to 0 ms pre-stimulus. Trials in which the participant had given an incorrect response were eliminated along with those contaminated by recording artefacts. Four participants had to be eliminated from further analysis because there were fewer than 16 segments in one or more of the conditions on either Day 1 or Day 2. The analyses are therefore based on data from 21 participants.

The remaining uncontaminated segments were averaged across participants for each electrode site. Grand average ERP waveforms were created separately for each of the four conditions; trained English words, trained novel words, untrained English words, and untrained novel words. On Day 1 the mean number of trials (range in brackets) contributing to the grand average ERP waveforms per participant were 24.05 (19–28) for trained English words, 24.24 (16–28) for trained novel words, 23.14 (19–28) for untrained English words, 24.52 (18–28) for untrained novel words. On Day 2 the mean numbers of trials were 23.76 (16–28) for trained English words, 24.24 (18–27) for trained novel words, 23.67 (17–28) for untrained English words, 24.62 (20–28) for untrained novel words. Three frontal (F3, Fz, F4), three central (C3, Cz, C4) and three parietal (P3, Pz, P4) electrode sites were selected for statistical analysis to allow for comparison of training effects according to coronal plane and laterality.

## Results

### Behavioural data

Error rates and reaction times were analysed only for participants who were included in the ERP analyses (N = 21, see next session). Incorrect responses were excluded from the reaction time analysis. Both reaction times and error rates were analysed using 2 x 2 x 2 repeated measures ANOVA, with Day (Day 1 vs. Day 2), training (trained vs. untrained) and word type (English word vs. Novel word) as within subjects factors.

#### Errors

The mean percentage of errors made on Day 1 and Day 2 in each of the four conditions are displayed in table 1. Overall error rates were very low with participants making an average of 1.83% errors on Day 1 and 2.72% errors on Day 2. There was a main effect of word type, *F*(1, 20) = 7.353, *MSE* = 0.992, *p* = 0.013, in that participants made more errors when responding to English words than when responding to novel words. There was also a significant interaction between training and word type, *F*(1, 20) = 9.486, *MSE* = 1.632, *p* = 0.006. A follow up analysis revealed that for novel words participants tended to make more errors in response to trained items, *F*(1, 20) = 5.268, *MSE* = 0.732, *p* = 0.033, whereas for English words participants made more errors in response to untrained items, *F*(1, 20) = 7.049, *MSE* = 1.839, *p* = 0.015. No other significant effects were present in the error rates.

**Table 1:** Mean percentage of errors on Day 1 and Day 2 of the Episodic Memory task (standard deviations in parenthesis).

Day 1				Day 2		
	English Words	Novel Words	Mean	English Words	Novel Words	Mean
Trained Items	1.18 (0.58)	1.71 (0.81)	1.45	2.04 (0.81)	2.89 (1.36)	2.47
Untrained Items	3.93 (1.30)	0.50 (0.48)	2.22	4.93 (1.76)	1.04 (0.56)	2.99
Mean	2.56	1.11	**1.84**	3.49	1.97	**2.97**

#### Reaction times

Table 2 displays the mean reaction times in response to words in each of the four conditions on Day 1 and Day 2. A significant main effect of Day was observed, *F*(1, 20) = 14.729, *MSE  = *18943.195, *p* = 0.001, indicating that participants were faster to respond on Day 2 in comparison to Day 1. A significant main effect of word type was also observed, *F*(1, 20) = 12.067, *MSE  = *11517.214, *p* = 0.002, in that participants responded faster to novel words than to English words. The main effect of training was also significant, *F*(1, 20) = 7.396, *MSE  = *10766.369, *p* = 0.013, in that participants were generally faster to confirm recognition of trained items than they were to reject untrained items. However, there was a significant interaction between training and word type, *F*(1, 20) = 50.808, *MSE  = *5183.665, *p*<0.001. Follow up analyses and inspection of the mean values in Table 1 indicates that for English words participants were faster to respond to trained items than to untrained items *F*(1, 20) = 38.121, *MSE  = *8297.431, *p*<0.001. For novel words in contrast, participants tended to be faster at rejecting untrained items than at recognising trained items, *F*(1, 20) = 3.487, *MSE*  = 7652.602, *p* = 0.077. Finally, there was a marginally significant interaction between Day and Word Type, *F*(1, 20) = 3.904, *MSE*  = 3608.304, *p* = 0.062. Follow up analyses and inspection of the mean values presented in Table 1 indicates that although participants tended to respond faster on Day 2 to both English words, *F*(1, 20) = 5.569, *MSE*  = 15056.256, *p* = 0.029, and novel words, *F*(1, 20) = 27.917, *MSE*  = 7495.244, *p*<0.001, the effect was greater for nonwords.

**Table 2:** Mean reaction times (reported in milliseconds) on Day 1 and Day 2 of the Episodic Memory task (standard deviations in parenthesis).

Day 1				Day 2		
	English Words	Novel Words	Mean	English Words	Novel Words	Mean
Trained Items	821 (99)	861 (119)	841	757 (85)	761(90)	759
Untrained Items	943 (137)	825 (125)	884	881 (147)	725 (129)	803
Mean	882	843	**862**	819	743	**781**

### Electrophysiological data

**Figure 1:**
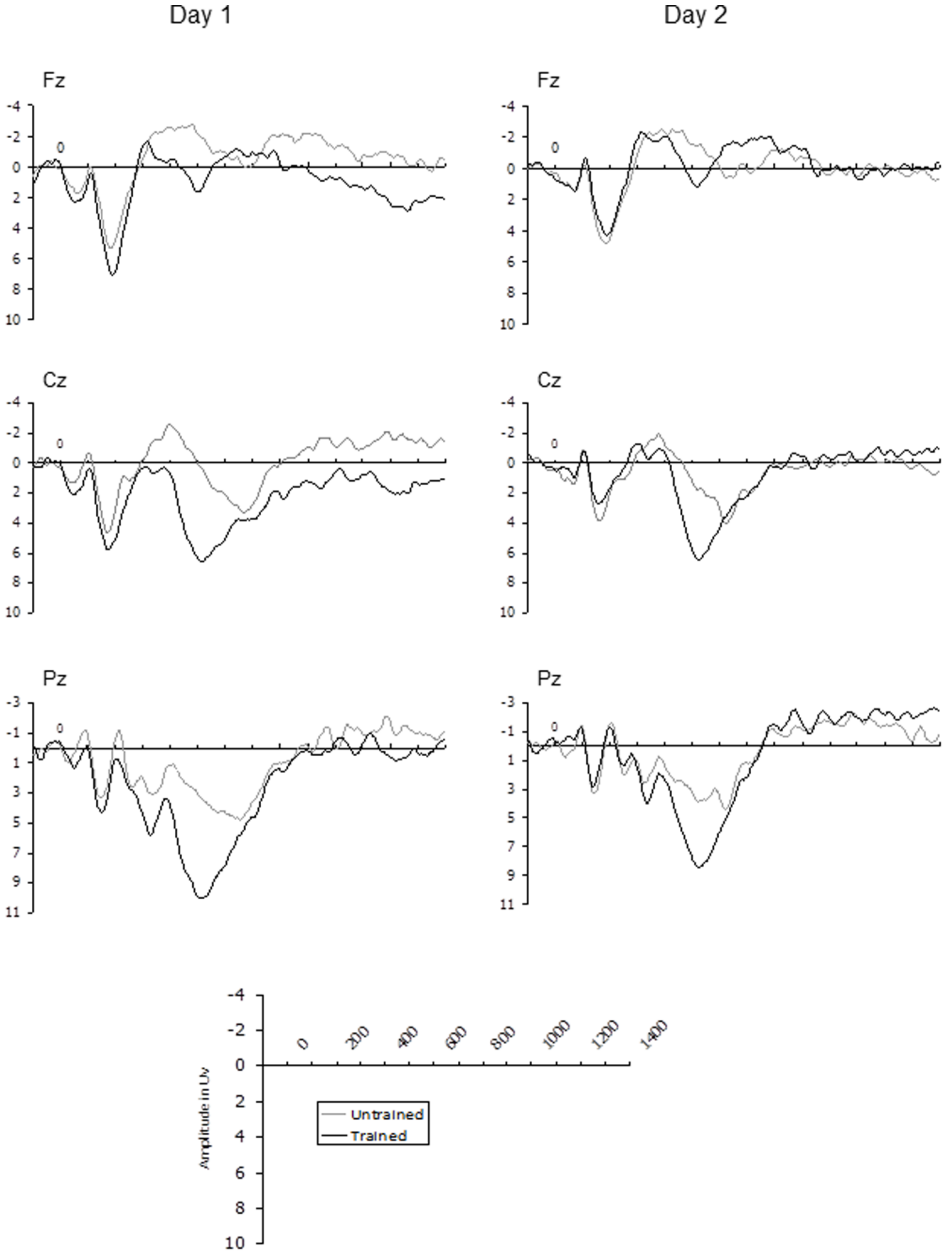
English Words: ERP responses to trained and untrained English words on day 1 and day 2.

**Figure 2:**
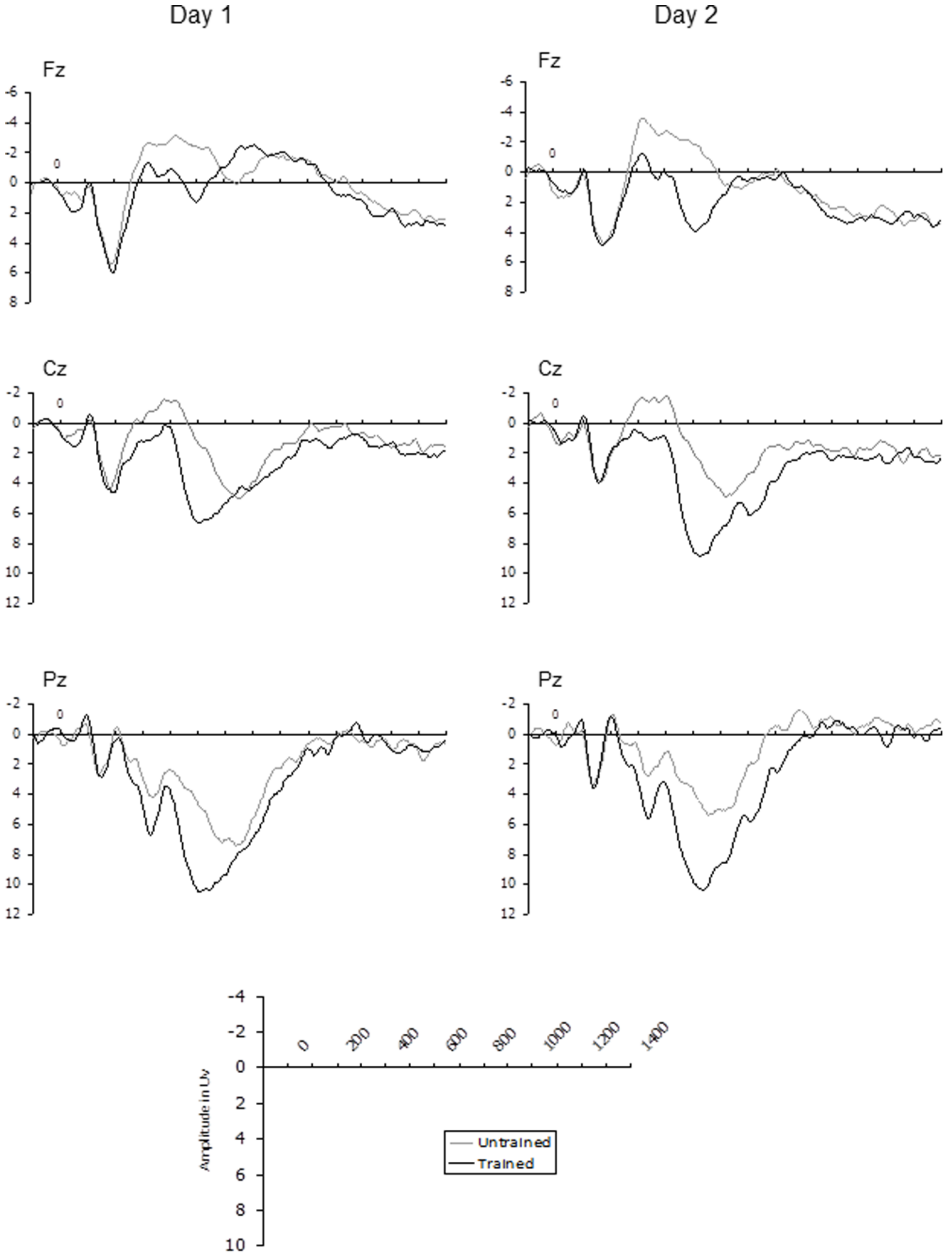
Novel Words: ERP responses to trained and untrained novel words on day 1 and day 2.

**Figure 3:**
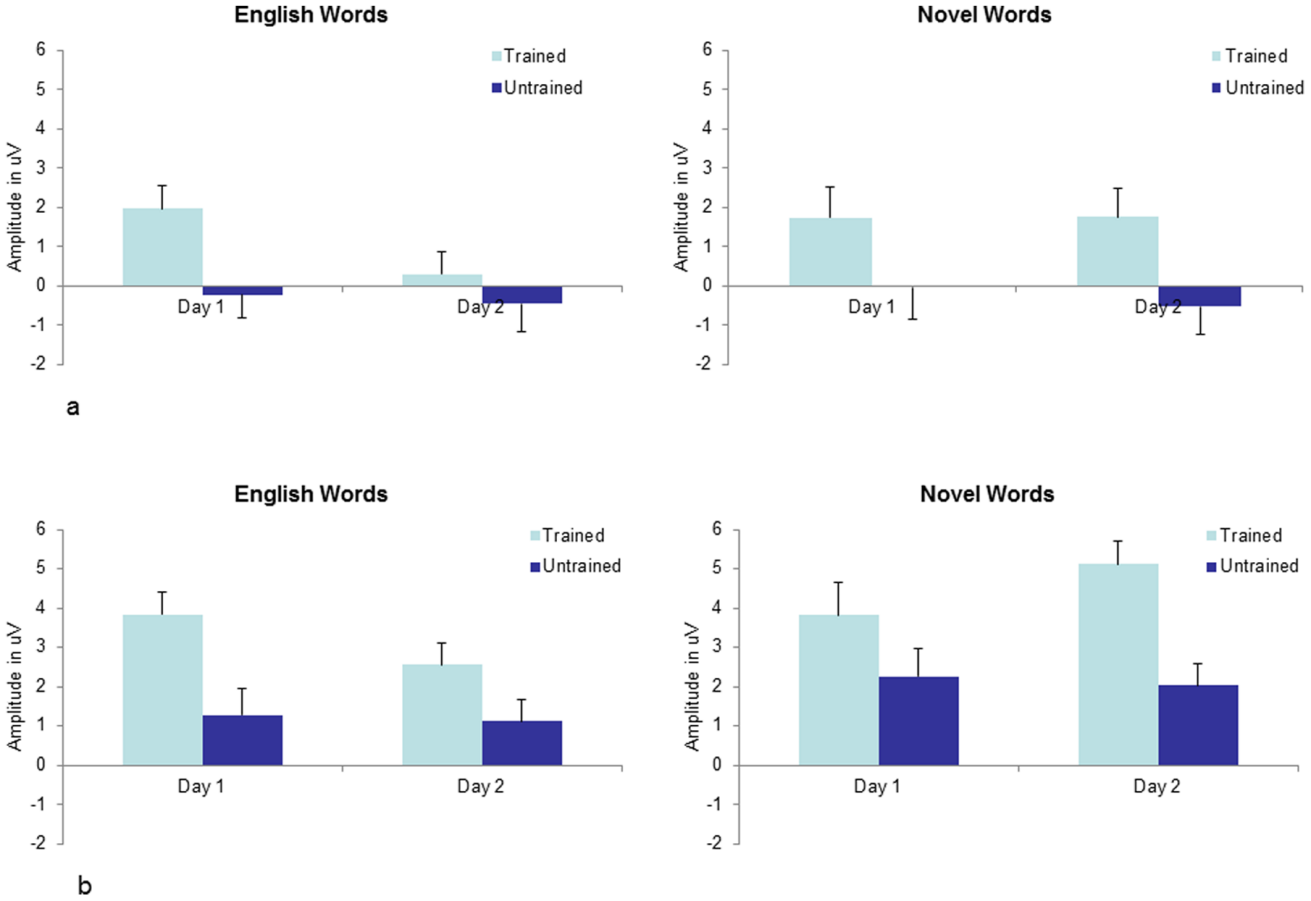
a Familiarity: Mean amplitude in the Familiarity (300–450 ms) time window collapsed across electrode sites (F3, Fz, F4, C3, Cz, C4, P3, Pz, P4). **b** Recollection: Mean amplitude in the Recollection (450–750 ms) time window collapsed across electrode sites (F3, Fz, F4, C3, Cz C4, P3, Pz, P4).

Grand average ERP waveforms representing training effects for English words and novel words at midline electrode sites (Fz, Cz, Pz) are shown in Figures 1 and 2 respectively. Clear effects of training can be observed in all of these ERP traces which extend from approximately 300 to 750 ms post-stimulus. In order to examine whether the effect of training differed between days, two time windows were selected for statistical analysis; 300–450 ms and 450–750 ms. These time windows seemed to best capture the observable differences in the ERP waveforms (see Figures 1 and 2) and were chosen to accord as much as possible with the established ERP old/new effects, consisting of an early component thought to reflect familiarity and a later component believed to represent recollection (9). The mean amplitude of both time windows were initially analysed using a 3×3×2×2×2 repeated measures ANOVA, with coronal plane (frontal vs. central vs. parietal), laterality (right vs. midline vs. left), training (trained vs. untrained items), Word Type (English words vs. Novel words) and Day (Day 1 vs. Day2) as within subjects factors. Follow up ANOVA then examined effects associated with English and novel words separately. The Greenhouse-Geisser [24] correction was applied to all ERP effects which had more than one degree of freedom in the numerator. In these instances an adjusted *p* value is reported along with the unadjusted degrees of freedom and Epsilon value. Mean values of these analyses are displayed in Figure 3a (300–450 ms time window) and Figure 3b (450–750 ms time window).

**Figure 4:**
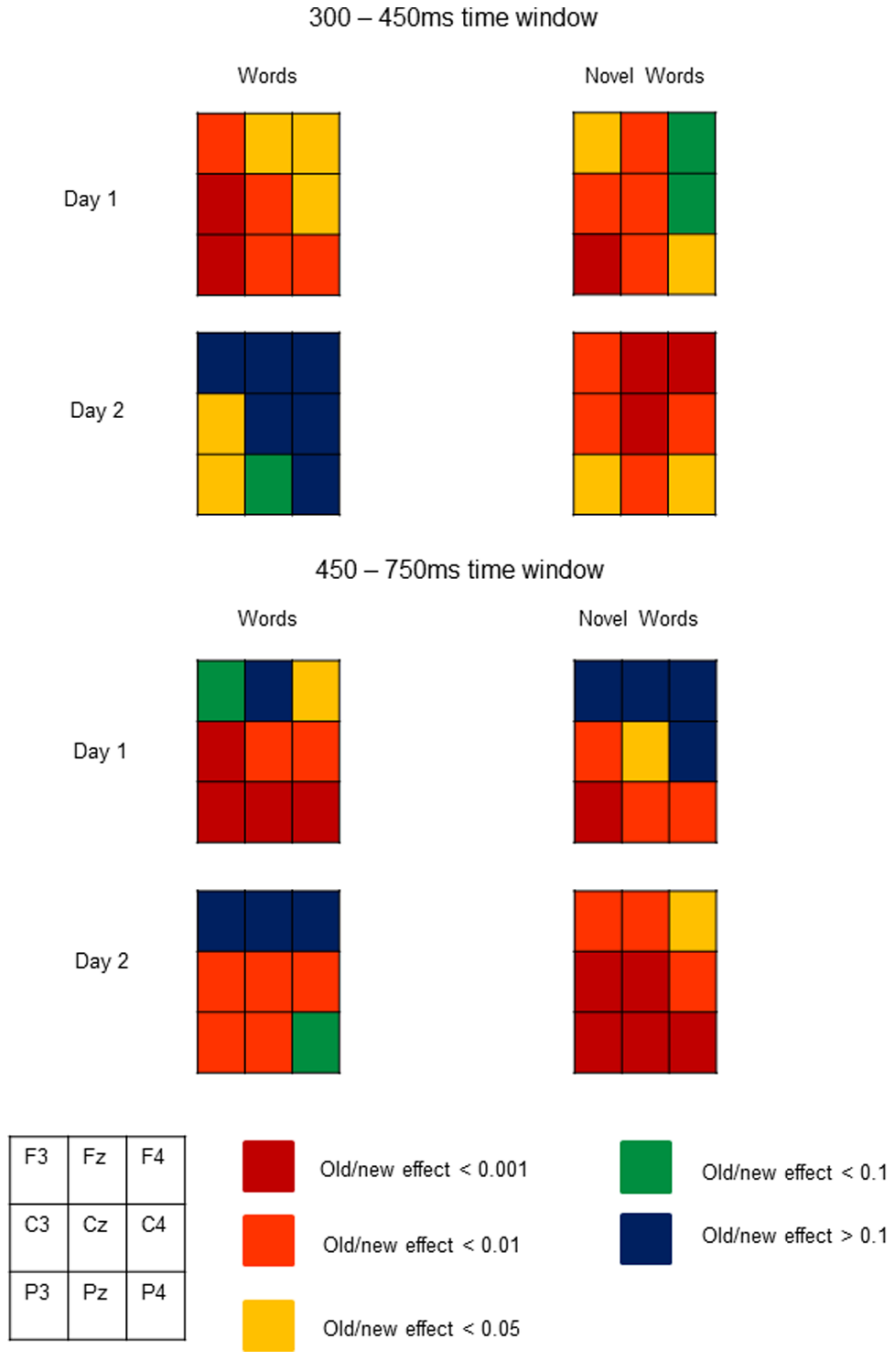
T-test maps showing the distribution of old/new effects for words and novel words on day 1 and day 2.

The distribution of effects is displayed in figure 4 which shows t-test outcomes for old/new comparisons at each of the nine electrodes.

#### 300–450ms window

Within the 300–450 ms time window the three way interaction between Training, Word Type and Day did not reach significance, *F*(1, 20) = 2.807, *MSE*  = 31.315, *p* = 0.109. However, there was a significant interaction between Day and Word Type, *F*(1, 20) = 4.450, *MSE*  = 10.521, *p* = 0.048, indicating that English and novel words elicited a similar mean amplitude on day 1, but not on day 2. In order to explore this interaction further, English words and novel words were analysed separately in two 3(Coronal Plane) ×3(Laterality) ×2(Training) ×2(Word Type) ANOVA.

For English words there was a significant main effect of training, *F*(1, 20) = 15.863, *MSE*  = 25.699, *p* = 0.001, confirming that trained items elicited more positive ERPs than untrained items in the 300–450 ms time window. There was also a marginally significant interaction between training and Day *F*(1, 20) = 4.161, *MSE*  = 22.828, *p* = 0.055. From inspection of Figure 3a it can clearly be seen that the effect of training was smaller on Day 2 in comparison to Day 1. Follow up analyses did indeed reveal that the main effect of training was significant on Day 1, *F*(1, 20) = 14.781, *MSE*  = 30.319, *p* = 0.001, but not on Day 2, *F*(1, 20) = 2.995, *MSE*  = 18.209, *p* = 0.10. On Day 1 there was a significant interaction between coronal plane and training *F*(2, 40) = 5.218, *MSE*  = 3.130, *p* = 0.022, ε = 0.673, indicating that the effect of training was slightly larger at parietal electrode sites. There were no other significant interactions on Day 1 or Day 2.

For novel words there was also a significant main effect of training in the 300–450 ms time window, *F*(1, 20) = 30.156, *MSE*  = 25.528, *p*<0.001, confirming that trained items elicited more positive ERPs than untrained items. The interaction between Training and Day was not significant, *F*(1, 20) = 0.544, *MSE*  = 22.674, *p* = 0.469, revealing that the early old/new effect typically associated with familiarity was similar on both days (see also Figure 3a). There was no significant interaction between training and coronal plane, *F*(2, 40) = 0.131, *MSE*  = 2.234, *p* = 0.877, ε = 0.694. The interaction between training and laterality was significant, *F*(2, 40) = 9.386, *MSE*  = 0.872, *p* = 0.001, ε = 0.938, indicating that the effects of training were largest at midline electrode sites.

#### 450–750ms window

Within the 450–750 time window there was a significant three way interaction between Training, Word Type and Day, *F*(1, 20) = 6.478, *MSE*  = 25.107, *p* = 0.019. Once again the interaction was followed up by examining effects English and novel words in separate ANOVA.

For English words there was a significant main effect of training in the 450–750 ms time window, *F*(1, 20) = 32.524, *MSE*  = 23.171, *p*<0.001, confirming that trained items elicited more positive ERPs than untrained items. The interaction between Training and Day was not significant, *F*(1, 20) = 2.042, *MSE*  = 28.219, *p* = 0.168. However, Planned Comparisons comparing the ERP response to trained items across days revealed a significant reduction in the amplitude of the late component on day 2 in comparison to day 1, *F*(1, 20) = 5.081, *MSE*  = 29.979, *p* = 0.036. A similar analysis comparing untrained items across days revealed no change in mean amplitude for either English words, *F*(1, 20) = 0.082, *MSE*  = 31.331, *p* = 0.777, or novel words, *F*(1,20) = 0.095, *MSE*  = 47.290, *p* = 0.761. There was also a significant interaction between training and coronal plane, *F*(2, 40) = 15.272, *MSE*  = 10.561, *p*<0.001, ε = 0.581, with the effects of training being largest at parietal electrode sites. The interaction between training and laterality was not significant, *F*(2, 40) = 0.334, *MSE*  = 3.946, *p* = 0.718, ε = 0.877.

For novel words the main effect of training was again significant, *F*(1, 20) = 22.780, *MSE*  = 44.784, *p*<0.001, indicating that trained items elicited more positive ERPs than untrained items. This time there was also a significant interaction between Training and Day, *F*(1, 20) = 5.035, *MSE*  = 21.664, *p*<0.036. Follow up analyses revealed that the main effect of training was significant on both Day 1, *F*(1, 20) = 5.619, *MSE*  = 41.121, *p* = 0.028, and Day 2 *F*(1, 20) = 35.465, *MSE*  = 25.327, *p*<0.001. However, as can be seen in Figure 3b, the interaction arose because the main effect of training for novel words was stronger on Day 2 in comparison to Day 1. On Day 1 there was a significant interaction between training and coronal plane, *F*(2, 40) = 31.847, *MSE*  = 3.083, *p*<0.001, ε = 0.695, with training effects being largest at parietal sites. There was no interaction between training and laterality, *F*(2, 40) = 2.626, *MSE*  = 3.412, *p = *0.102, ε = 0.752. On Day 2 training continued to interact with coronal plane, *F*(2, 40) = 8.378, *MSE*  = 5.217, *p* = 0.003, ε = 0.711, with largest effects again appearing at parietal sites. On Day 2 training also interacted with laterality, *F*(2, 40) = 4.671, *MSE*  = 2.500, *p* = 0.015, ε = 0.813, with training effects appearing largest at left and midline electrode sites.

## Discussion

The ERP results of the present study revealed a dissociation in the way in which recognition memory for familiar and novel words changed over time. This difference was evident primarily in the 450–750 ms time window associated with recollection. In all instances, the change in the ERP old/new effects that occurred across days was driven entirely by a shift in response to trained items while responses to untrained items were unaffected. These changes are discussed in detail below. In terms of behavioural responses, there was no significant change in overall accuracy rates across days, most likely due to the extensive training programme creating strong memory traces from the start. Response times to all stimuli decreased on Day 2, presumably because participants had practice at performing the recognition task.

### Familiar English words

For familiar English words, ERPs occurring 300–450 ms post stimulus onset distinguished trained from untrained items on Day 1, but not on Day 2. Given that all English words were known by participants prior to training, and therefore were familiar to some extent, it is likely that familiarity was simply less useful in distinguishing trained from untrained items, particularly on Day 2. Indeed, this would be supported by the fact that the typical frontal maximum was not observed for the early ERP old/new effect for English words on both days (see Figure 4, note also that other cases in which the early old/new effect shows a parietal distribution have been reported in the literature [e.g. 25]). Participants therefore had to rely on explicit recollection to decide whether or not an item had appeared in the training phase [12], [26]. During the recollection time window (450–750 ms) ERPs distinguished trained from untrained items on both days, but the amplitude of the LPC in response to trained items was reduced on Day 2 in comparison to Day 1. This suggests that the memory traces underpinning recollection for familiar English words weakened over time.

It should be noted that the English words used in this experiment came from only 4 semantic categories, and therefore some spreading activation, or priming of semantic relatives may have occurred during the training phase. Since the untrained English words that appeared during the recognition task were drawn from the same 4 semantic categories as the trained words, rejecting untrained English words may have been more difficult than rejecting untrained novel words. Indeed the behavioural data indicates that for English words participants were faster and more accurate at responding to trained items, whereas for novel words participants were faster and more accurate at responding to untrained items. Similarly, one might argue that this spreading activation is partially responsible for the weakening of ERP old/new effects on Day 2. However, although this may have contributed to the observed effects we believe that this is not the most plausible explanation for a number of reasons. Firstly, there was virtually no change in the ERP response to untrained items across days (see Figure 3). If the ERP results could be explained by the fact that participants experienced more difficulty in rejecting untrained items on Day 2, it does not clearly follow that only the response to *trained* items should shift. There is therefore no evidence in the ERP data to suggest that untrained items were more familiar on the second day. Secondly, since participants underwent a very extensive training phase, the error rate on Day 2 was not significantly higher than the error rate on Day 1, indicating that participants were not ‘forgetting’ which items they had learned. Note that in Joyce and Kutas' [19] study the error rate was very high (less than 50% hits the day after training), yet they still observed an increase in the old/new effect. We propose therefore that weakening of the ERP recognition response to familiar English words was primarily due to trace decay rather than interference.

### Novel words

For novel words there was no interaction between Training and Day in the early (300–450 ms) time window indicating that the magnitude of this component was similar across days. It appears therefore that the novelty of these items meant that they were able to retain their familiarity effect over time. Indeed it is worth noting that, unlike for English words, early old/new effects for novel words tended to show the more traditional frontal distribution typically associated with familiarity, particularly on day 2 (see figure 4).

More interesting however, is the fact that the trained versus untrained difference in the recollection (450–750 ms) time window became significantly *larger* on Day 2 in comparison to Day 1, despite the fact that participants had received no additional training. Once again this was driven entirely by an increase in positivity in response to trained items, as the ERP response to untrained items remained the same across days. This suggests that some form of consolidation for novel material may have occurred over time, leading to stronger recollection on Day 2. This finding is consistent with the results of Joyce and Kutas [19] who also observed a larger old/new difference in the LPC the day after participants had been trained on novel faces. In Joyce and Kutas' study however, no old/new effects were observed on the same day as training. A possible reason for this discrepancy is that our training phase was considerably more extensive and participants were trained on fewer items which may have allowed stronger representations to form in long term memory during training. Importantly, the present study indicates that even when participants have received very extensive training and behaviourally perform at ceiling level across testing sessions, effects of consolidation can still be observed in ERP data.

### General discussion

Overall, the results of the present study demonstrate a dissociation in the way in which recognition memory for trained familiar and novel words changes over time. In both the early and late time windows, the ERP response to trained novel words and trained English words was very similar on Day 1. This indicates that the training phase was successful in ensuring that English and Novel words were learned to a similar standard and recognised equally well immediately after training. Divergence in the ERP response to trained items only occurred after a period of consolidation, indicating weakening in recognition memory for trained familiar words and strengthening in memory for trained novel words. This difference in the ERP responses occurred despite the fact that accuracy rates for both familiar and novel words remained extremely high and did not differ significantly across days. Furthermore, in contrast to the ERP data, behavioural responses to trained English and trained novel words were very similar on Day 2. This highlights the fact that relying on behavioural responses alone may sometimes be misleading when examining effects of memory consolidation.

The conclusion that consolidation effects are stronger for novel information is intuitive and most likely reflects an adaptive process as it is more important to retain novel information or experiences than those that are already familiar. It is also consistent with the findings of a number of other studies. In an early experiment, Salasoo et al. [27] observed that recognition memory for pseudowords was superior to that for words following a delay interval of one year. Salasoo et al. commented that this result was somewhat surprising given that pseudowords were not recognised very well on immediate recognition tests. More recently, a study by Melendez et al. [28] which set out to test the effect of hypnotics on sleep induced memory improvement found no difference between treatment groups. They did note however, that participants in the sleep condition recalled more nonwords than those in a wake comparison group, but there was no significant between group difference in the number of standard words recalled.

Results of this nature, including ours, may be linked to the fact that novel information is more likely to undergo significant representational change as it becomes integrated with existing knowledge. In the context of novel word learning, a number of studies by Gaskell and colleagues [e.g. 29, 30] have demonstrated that novel word forms only engage in lexical competition with existing word forms after a period of consolidation. Interestingly sleep seemed to be a necessary requirement for novel word forms to become fully lexicalised, since no competition effect were observed after an equivalent period of wakefulness. Since the present study did not include a wake comparison, it cannot be confirmed that the observed effects occurred as a direct result of sleep or whether they are simply associated with the passage of time. However, there is substantial evidence that newly acquired memory representations are reactivated in the hippocampus during slow wave sleep (SWS) [e.g. 31, 32] which may facilitate explicit recollection of these memories [11].

The results of the present study are also broadly consistent with behavioural studies which indicate that sleep benefits recollection but not familiarity [10], [11]. It should be noted that both of these previous studies included only existing (i.e. familiar) words as stimuli, and in the study by Daurat et al. [10] sleep was associated with a reduction in the proportion of loss over time compared to when the retention period was filled with wakefulness. In the study by Drosopoulos et al. [11] no recognition test was conducted immediately after the study phase, and so it is not known how memory changed over time. Nevertheless, the results of these behavioural studies converge with the ERP results of the present study in providing evidence that recollection is most likely to benefit from consolidation, and suffers a smaller decline over time than familiarity.

Interestingly, although early ERP effects in response to English words disappeared on Day 2, they were maintained for novel words. As pointed out by Yovel and Paller [33], known words have a high baseline familiarity as they are associated with information from many pre-experimental episodes. The consequence may be that over time familiarity becomes less useful in distinguishing between studied and unstudied words. It is likely that this occurs because it becomes increasingly difficult to distinguish between the study episode and pre and post-experimental experiences with a known word on the basis of familiarity. To perform the recognition task the participant is therefore dependent upon their ability to reinstate the context of the study episode. Since novel words have only been seen in the context of the study, the same logic does not apply, and familiarity will continue to contribute to recognition decisions for these items. This finding therefore supports the argument made by Yovel and Paller [33] that known words may not make ideal stimuli for studying the processes of recognition memory.

Finally, it should be noted that in the present study, the same stimuli were used in the recognition task on both Day 1 and Day 2. This was necessary because the number of stimuli used in the experiment was restricted by the number of items that participants could be expected to learn to a level of 100% accuracy in a single training session. One might argue therefore that the recognition task on Day 1 provided additional exposure to items which may have contributed to the larger recollection effects observed in the 450–750 ms time on Day 2. In relation to this point, we would like to highlight that the most important finding of this study, indicated by the significant three way interaction between Word Type, Training and Day in the 450–750 ms time window, is that there is a *dissociation* in the way in which the recollection response to familiar and novel words changes over time. Since both familiar and novel items were repeated across days, this dissociation cannot be explained by the fact that stimuli were repeated in the Day 1 and Day 2 tests. Secondly, our result relating to novel words is consistent with other studies which have not repeated the same items across days, but have still observed an increase in the late positive component in response to trained novel faces on the second day of testing [19]. Thirdly, in the present study all items were repeated across days yet the increase in the late positive component on Day 2 was observed only in response to trained novel words. The same untrained English and novel words were also used on both days, yet the ERP response was unaffected by the participant having seen these items during the Day 1 test. It seems therefore that mere exposure is not sufficient for consolidation to occur, but rather that it may be dependent on the participant making some level of conscious effort to encode items during training. This is consistent with recent research showing that consolidation during sleep occurs only when information is expected to be of future relevance [34]. Finally, it should be noted that no feedback was given during the day 1 test.

### Conclusions

In sum, this study yielded a number of interesting and important findings. Firstly the results demonstrate that even when participants receive extensive training on novel material and perform with a very high level of accuracy, evidence of consolidation can still be observed in response to items that are correctly remembered. This finding converges with data from fMRI studies which have shown that covert reorganisation of functional brain activity which occurs during sleep is not necessarily reflected in overt changes in behaviour [35]. Secondly, our data suggest that consolidation processes for novel and familiar material may be different. Despite similar performance on behavioural measures, in the ERP data recognition memory for familiar items showed a pattern of overall loss, whereas memory for novel items appeared to be enhanced over time. As information is continually acquired, memory must be constantly updated with new information. Since familiar words are already known, consolidation is directed towards novel information which is likely to undergo significant representational change as it becomes integrated with existing knowledge. Further research is needed to determine whether sleep plays any role in the observed dissociation.

## Supporting Information

Appendix S1 **Examples of novel words forms used in experiment 1.**
(DOCX)
