# Supplementary material for: Changes in Recognition Memory over Time: An ERP Investigation into Vocabulary Learning
Source: PLoS One. 2013 Sep 5;8(9):e72870. doi: 10.1371/journal.pone.0072870 (PMC3764170; doi:10.1371/journal.pone.0072870)
Supplement: Appendix S1 — Examples of novel words forms used in experiment 1. (DOCX) [file pone.0072870.s001.docx]

**Appendix 1**: Examples of novel words forms used in experiment 1

Bem Polbro Reb

Verk Carbella Dern

Torbs Gloach Jum

Kence Brab Vont

Rabilats Cule Casonore

Konth Serdu Chell

Churk Trunch Nart
